# Supplementary material for: The miR-141/200c-STAT4 Axis Contributes to Leukemogenesis by Enhancing Cell Proliferation in T-PLL
Source: Cancers (Basel). 2023 Apr 28;15(9):2527. doi: 10.3390/cancers15092527 (PMC10177500; doi:10.3390/cancers15092527)
Supplement: Supplementary file 1 [file cancers-15-02527-s001.zip › cancers-2338971-supplementary.pdf]

Supplementary Materials: The miR-141/200c-STAT4 Axis Contributes to Leukemogenesis by Enhancing Cell Proliferation in T-PLL

Moritz Otte, Johanna Stachelscheid, Markus Glaß, Linus Wahnschaffe, Qu Jiang, Waseem Lone, Aleksandr Ianevski, Tero Aittokallio, Javeed Iqbal, Michael Hallek, Stefan Hüttelmaier, Alexandra Schrader, Till Braun and Marco Herling

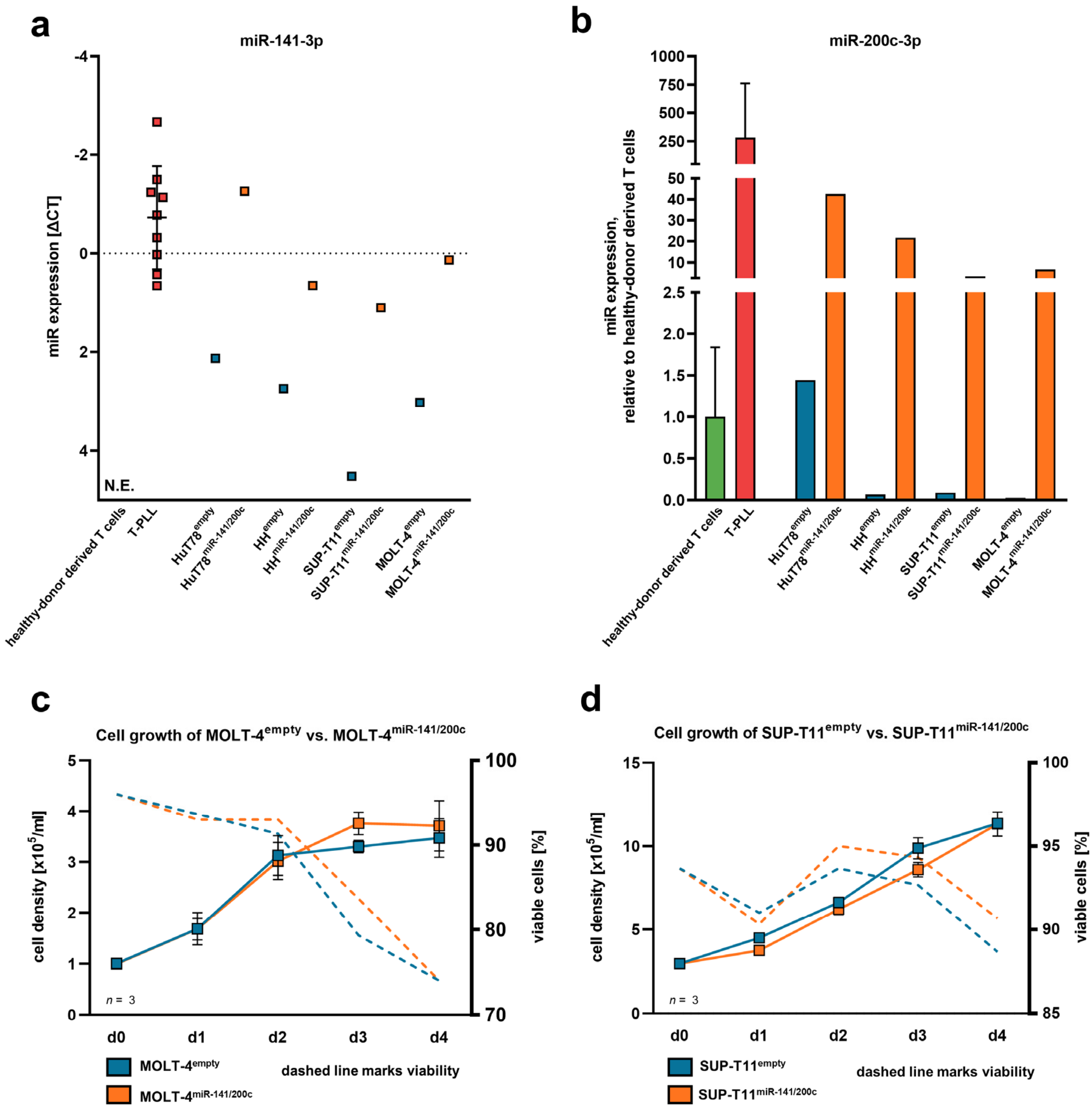

Figure S1. Overexpression of the miR-141/200c cluster has no impact on cell proliferation and cell death in the naive, T-ALL-like cell lines MOLT-4 and SUP-T11. Data supplementing Figure 2. To interrogate whether higher levels of miR-141/200c lead to a more aggressive and proliferative cellular phenotype, we introduced lentiviral vectors with or without the miR-141/200c genomic cluster into the naive T-acute lymphocytic leukemia (T-ALL) cell lines MOLT-4 and SUP-T11. (**a**,**b**) Expression of miR-141-3p and miR-200c-3p in healthy-donor derived T cells (n=4, green) and T-PLL (n=9, red) cells as well as in TCL cell lines with (miR-141/200c, orange) and without (empty, blue) experimentally introduced miR-141/200c expression (n=1 each) as analyzed by quantitative real-time PCR (qRT-PCR). Means with standard deviation (if applicable) are presented. (**a**) ΔCT values of miR-141-3p as analyzed by qRT-PCR. We did not detect any miR-141-3p in healthy-donor derived T cells.

Because of this, the expression is presented as ΔCT values. Lower or negative ΔCT values reflect a higher expression of the respective miR. N.E. = no expression. (**b**) Relative expression of miR-200c-3p as analyzed by qRT-PCR. Relative expression was calculated using the 2-ΔΔCT method, with the mean 2-ΔΔCT values of the 4 healthy-donor derived T-cell controls used as the reference. Notably, TCL cell lines showed significantly lower miR-141/200c expression as compared to primary T-PLL cells. Upon experimental miR-141/200c expression in these TCL cell lines, miR expression values of the TCL cell lines became similary to those of T-PLL samples, therefore, representing a suitable T-PLL-like cellular model for these purposes. (**c**,**d**) Cell density and viability as measured by Trypan blue staining of MOLT-4$^{miR-141/200c}$ and MOLT-4^{empty} cells (**c**), as well as of SUP-T11$^{miR-141/200c}$ and SUP-T11^{empty} cells (**d**). MOLT-4 and SUP-T11 cells with and without miR-141/200c overexpression were seeded at a low density (1x105 cells/mL) and cultured over four days in a culture medium with low serum contents (RPMI + 1% FBS). On each day, means with standard error of the mean (SEM) are presented. In contrast to the effects observed in the mature T-cell lymphoma lines HuT78 and HH, miR-141/200c upregulation did not significantly affect cell densities as well as cell viability (two-way ANOVA).

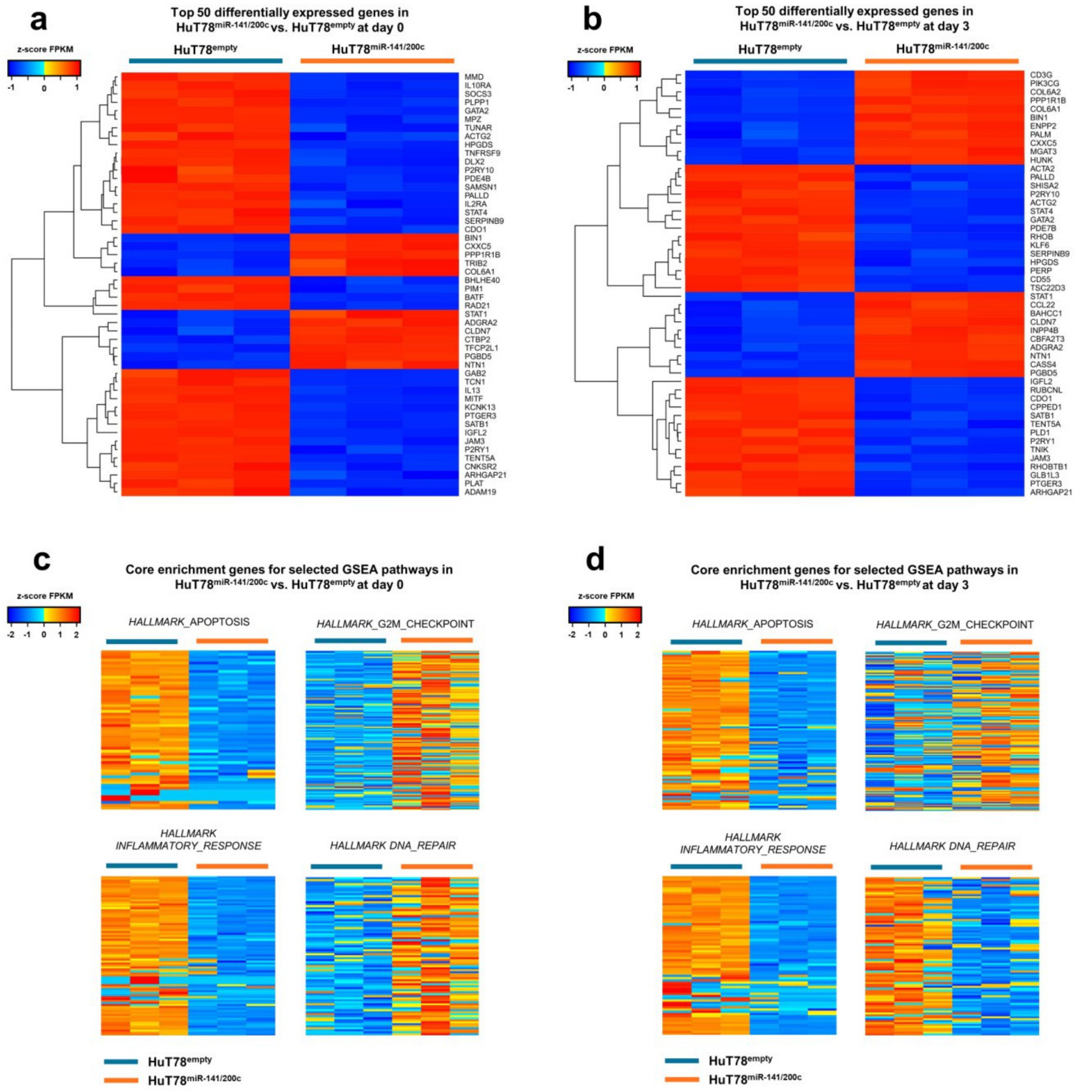

Figure S2. Overexpression of miR-141/200c shapes a pro-oncogenic transcriptome of the mature T-cell lymphoma line HuT78, affecting pathways of cell cycle regulation, DNA repair, and inflammatory responses. Data supplementing Figure 3. Analyses of differentially expressed genes comparing HuT78$_{miR-141/200c}$ vs. HuT78$_{empty}$ cells, without any cell culture effect (day 0 (d0)) and after three days of serum starvation (RPMI + 1% FBS; d3). For each time point, three technical replicates were sequenced for each transduced cell line. (**a**,**b**) Heatmap of the 50 highest differentially expressed mRNAs, comparing HuT78$_{miR-141/200c}$ cells to HuT78$_{empty}$ cells at d0 (**a**) and d3 (**b**). The presented colors represent z-scores of respective Fragments Per Kilobase Million (FPKM) values calculated for each mRNA (red=higher z-score; blue=lower z-score). (**c**,**d**) Heatmaps of core-enriched genes of

exemplary *HALLMARK* gene sets comparing HuT78$_{miR-141/200c}$ cells to HuT78$_{empty}$ cells at d0 (**c**) and d3 (**d**). The presented colors represent z-scores of respective Fragments Per Kilobase Million (FPKM) values calculated for each mRNA (red=higher z-score; blue=lower z-score). Upon miR-141/200c up-regulation, a specific gene expression signature is observed in HuT78 cells, affecting pathways of cell cycle regulation, DNA repair mechanisms, and inflammatory responses.

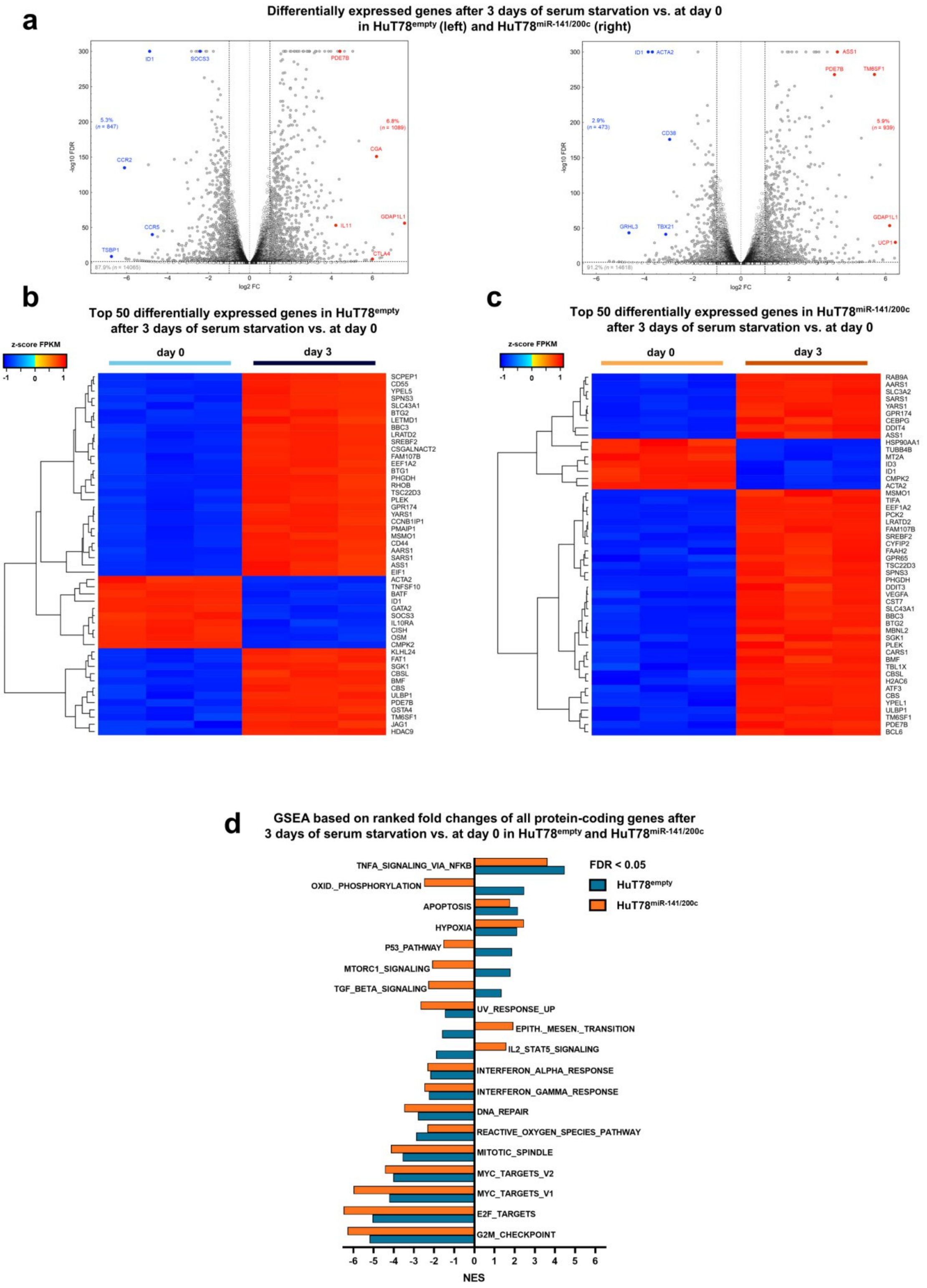

Figure S3. Serum starvation shapes the gene expression signature of HuT78 cells, showing aberrant cell cycle progression and enhanced apoptosis induction. Data supplementing Figure 3. Analyses of differentially expressed genes comparing HuT78$_{empty}$ cells without any cell culture effect (day 0 (d0)) with HuT78$_{empty}$ cells after three days of serum starvation (RPMI + 1% FBS; d3), as well as comparing HuT78$_{miR-141/200c}$ at d0 vs. d3. For each time point, three technical replicates were sequenced for each

transduced cell line. (**a**) Volcano plots of all expressed mRNAs in HuT78$_{empty}$ (left) and HuT78$_{miR\text{-}141/200c}$ cells (right), comparing d0 with d3. The horizontal dashed line indicates an FDR of 0.01. The black vertical dashed lines have an fc of 0.5 and 2; the light grey vertical line marks an fc of 1. Exemplary genes are highlighted in blue (downregulation) or red (upregulation). (**b**,**c**) Heatmap of the 50 highest differentially expressed mRNAs in HuT78$_{empty}$ (**b**) and HuT78$_{miR\text{-}141/200c}$ cells (**c**), comparing d0 with d3. The presented colors represent z-scores of respective Fragments Per Kilobase Million (FPKM) values calculated for each mRNA (red=higher z-score; blue=lower z-score). (**d**) GSEA (*HALLMARK* gene sets), based on the complete list of fold changes of all protein-coding genes comparing d0 with d3 (FDR<0.01, Kolmogorov-Smirnov test). The blue bars indicate differentially enriched *HALLMARK* gene sets in HuT78$_{empty}$ cells and the orange bars in HuT78$_{miR\text{-}141/200c}$ cells. We observed a specific transcriptome upon serum starvation, impacting pathways of T-cell receptor (TCR) signaling, inflammatory responses, cell cycle regulation, and apoptosis induction. Supplementary Table 4 summarizes differential gene expression and deregulated GSEA pathways comparing transcriptomes at d0 and d3 for either HuT78$_{empty}$ or HuT78$_{miR\text{-}141/200c}$ cells.

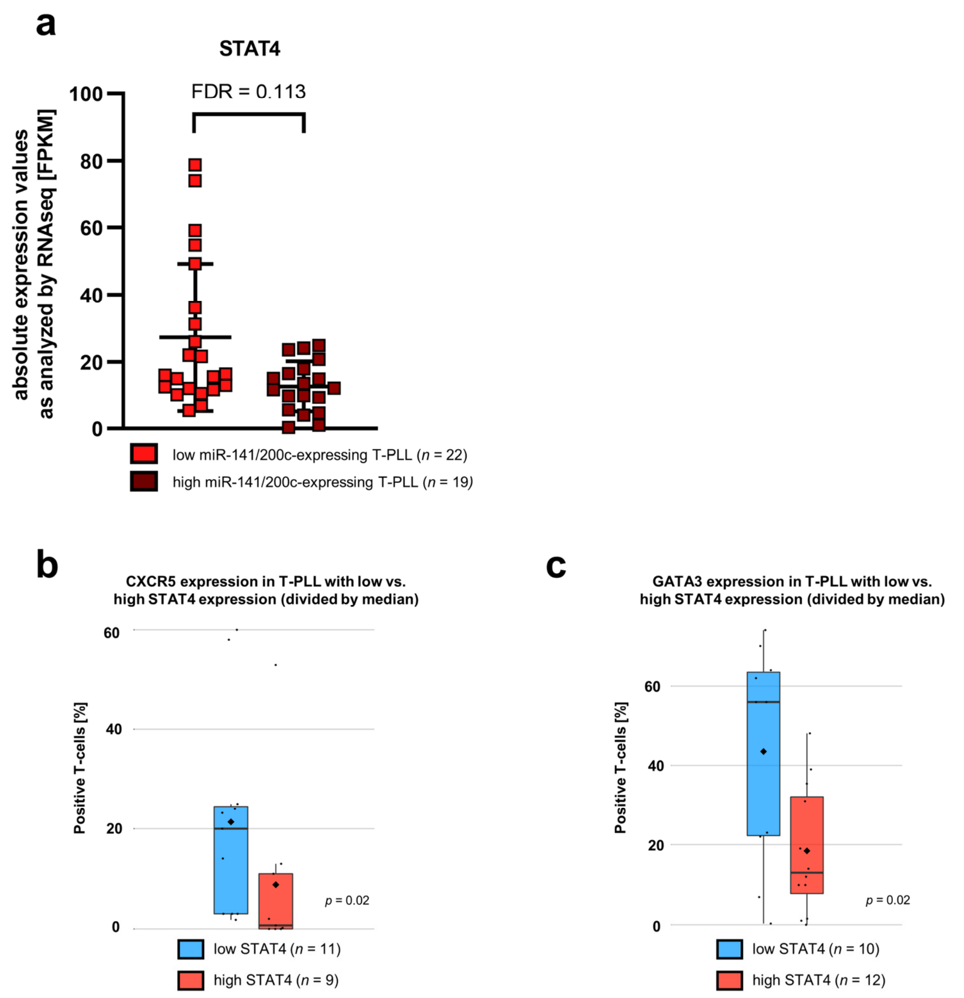

Figure S4. High miR-141/200c T-PLL possess lower *STAT4* expression, which is associated with elevated CXCR5 and GATA3 surface expression in primary T-PLL cells. Data supplementing Figure 4. (**a**) Absolute expression values of STAT4 mRNA as analyzed by RNA-seq in high (n=19, dark red) vs. low (n=22, light red) miR-141/200c expressing T-PLL cases. STAT4 expression was lower in high miR-141/200c expressing T-PLL compared to cases with low miR-141/200c expression. These differences were not significant when correcting for multiple testing (fc=0.464, p=0.002, FDR=0.113). (**b**,**c**) Association analyses of *STAT4* mRNA expression with CXCR5 (**b**) and GATA3 (**c**) surface expression in primary T-PLL cells. Groups of low and high *STAT4* expression were assigned by results of GEP array analyses[4] and divided by median: after division into two groups, cases with lower (blue) were compared to those with higher (red) *STAT4* expression. Lower levels of *STAT4* mRNA were associated with a higher number of malignant cells expressing CXCR5 and GATA3.

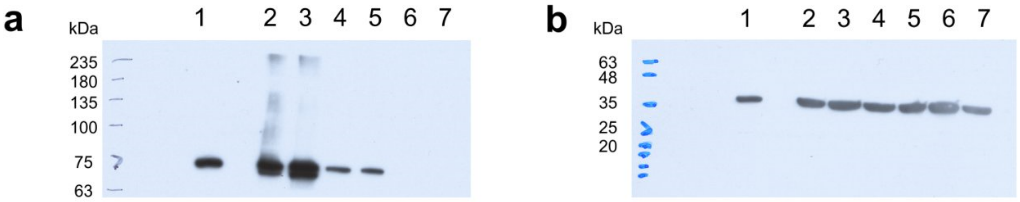

Western blot using anti-STAT4 (cs-2653, Cell Signaling Technology, panel **(a)**) and anti-β-Actin (sc-1616, Santa Cruz Biotechnology, panel **(b)**) antibody in healthy-donor derived T cells and primary T-PLL.
loading order:
1) NK-92 (positive control); 2,3) healthy-donor derived T-cell control 1 and 2; 4, 5) low miR-141/200c T-PLL 1 and 2; 6, 7) high miR-141/200c T-PLL 1 and 2. No signal for STAT4 in lanes 6 and 7.

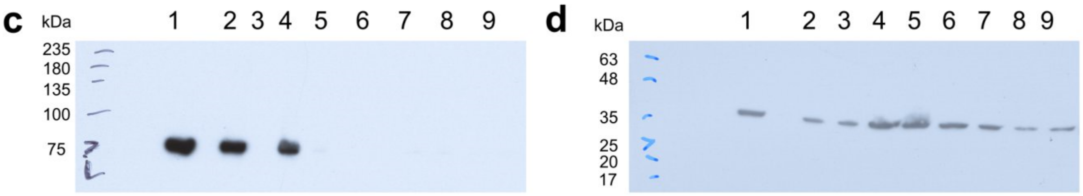

Western blot using anti-STAT4 (cs-2653, Cell Signaling Technology, panel **(c)**) and anti-β-Actin (sc-1616, Santa Cruz Biotechnology, panel **(d)**) antibody in T-cell leukemia/lymphoma lines with and without enforced miR-141/200c overexpression.
loading order:
1) NK-92 (positive control); 2) HuT78^{empty}; 3) HuT78$^{miR-141/200c}$; 4) HH^{empty}; 5) HH$^{miR-141/200c}$;
6) SUP-T11^{empty}; 7) SUP-T11$^{miR-141/200c}$; 8) MOLT-4^{empty}; 9) MOLT-4$^{miR-141/200c}$. No signal for STAT4 in lanes 3, 5-9.

Figure S5. Complete scans of immunoblots for STAT4 and β-Actin. Data supplementing Figure 4. Uncropped immunoblots on whole protein lysates of primary T-PLL, healthy-donor derived CD3+ T cells, and T-cell leukemia/lymphoma lines. The primary antibodies used were STAT4 (cs-2653; Cell Signaling Technology, Danvers, USA) and β-Actin (sc-1616; Santa Cruz Biotechnology, Dallas, Texas) at a 1:1000 dilution. The markers for molecular weight [kDa] can be seen on the left of each scan.

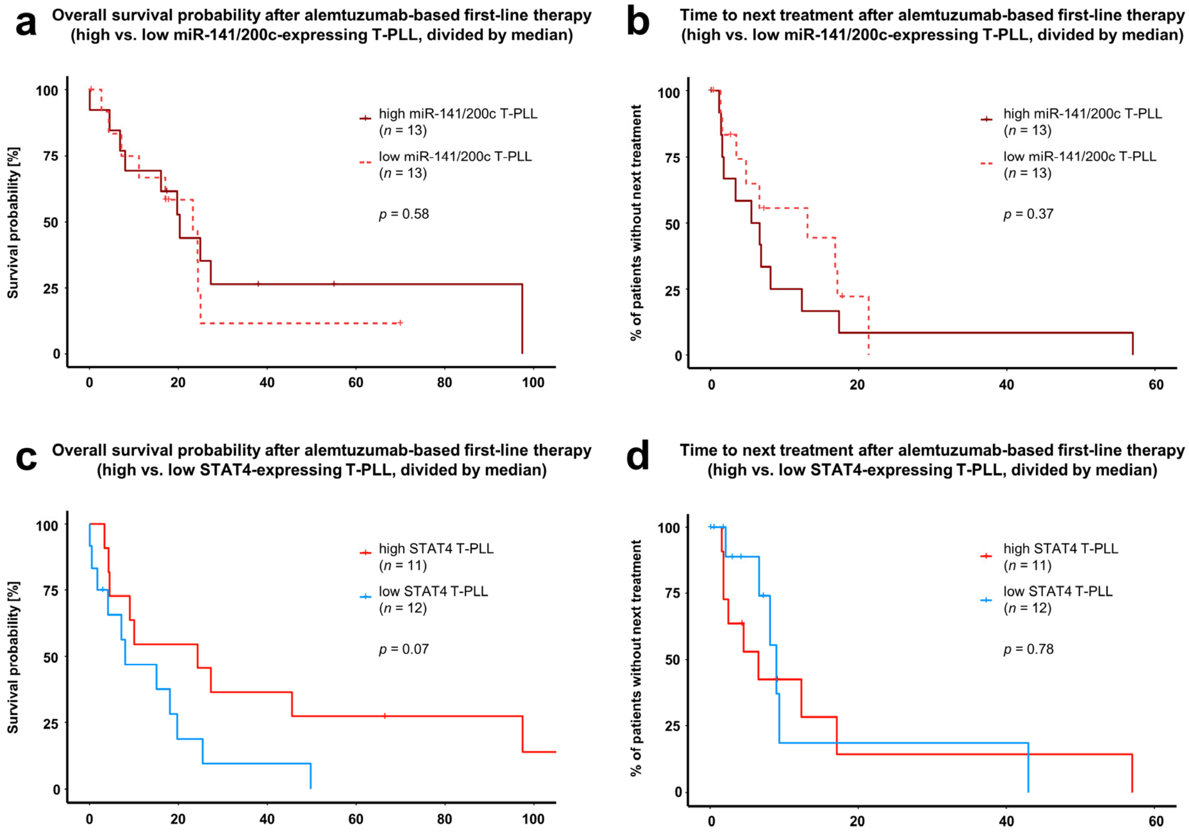

Figure S6. Outcomes after alemtuzumab-based first line therapy in T-PLL in association with miR-141/200c or *STAT4* expression. Outcomes following alemtuzumab-containing induction therapy (49/70 (70%) of entire cohort) as assessed by overall survival (OS) probability upon treatment (displayed in (**a**) and (**c**)) or by time to next treatment (TTNT, defined as time from index date of first treatment to the initiation of a next treatment, displayed in (**b**) and (**d**)). Cases were divided by the median miR-141/200c expression ((**a**) and (**b**), light red, dotted line = low miR-141/200c, dark red = high miR-141/200c, as analyzed by small-RNA sequencing) or *STAT4* expression ((**c**) and (**d**), blue = low *STAT4*, red = high *STAT4*, as analyzed by gene expression arrays) of all T-PLL cases. Statistical significance was assessed using the log-rank test.

Table S1. Clinical, cytogenetic, and immunophenotypic characteristics of involved T-PLL cases.

**Patient characteristics**		
Median age at diagnosis, years (range)[1]	68 (32-88); *n* = 91	
Sex	Male = 57<br>Female = 37	
Median OS from diagnosis, months (range)[1]	24.4 (0.4-129.9); *n* = 90	
**Clinical presentation**	**At diagnosis**	**At sample**
Median WBC count, x10⁹/L (range)[1]	51.4 (9.9-825.2); *n* = 73	91.12 (5.3-756.2); *n* = 88
Median hemoglobin, g/dL (range)[1]	12.9 (6.6-18.1); *n* = 65	12.6 (6.2-15.8); *n* = 71
Median platelet count, x10⁹/L (range)[1]	167.5 (33-438); *n* = 66	121 (9-506); *n* = 71
Median LDH, U/L (range)[1]	502 (178-9423); *n* = 49	639 (168-8634); *n* = 59
Splenomegaly (%)[2]	*n* = 38/65 (58.5)	
Hepatomegaly (%)[2]	*n* = 11/62 (17.7)	

Lymphadenopathy (%)[2]	*n* = 42/64 (65.6)	
**Genomic lesions (cytogenetics)**		
inv(14)(q11;q32) (%)[2]	*n* = 44/67;	(65.7)
t(14;14)(q11;q32) (%)[2]	*n* = 7/67;	(10.4)
t(X;14)(q28;q11) (%)[2]	*n* = 5/67;	(7.5)
TCR gene rearrangement[3] (%)[2]	*n* = 63/70;	(90.0)
MYC amplification[3] (%)[2]	*n* = 43/53;	(81.1)
ATM deletion[3] (%)[2]	*n* = 36/60;	(60.0)
**Immunophenotype**		
TCL1 (%)[2]	*n* = 76/87;	(87.4)
$CD3^+$ (%)[2]	*n* = 81/90;	(90.0)
$CD5^+$ (%)[2]	*n* = 87/88;	(98.9)
$CD7^+$ (%)[2]	*n* = 85/87;	(97.7)
$CD4^+/CD8^-$ (%)[2]	*n* = 61/90;	(67.8)
$CD4^-/CD8^+$ (%)[2]	*n* = 13/90;	(14.4)
$CD4^+/CD8^+$ (%)[2]	*n* = 15/90;	(16.7)
$CD4^-/CD8^-$ (%)[2]	*n* = 1/90;	(1.1)

number of available data sets of our patient cohort is reflected by *n* for each parameter; [1] range reaches from lowest value to highest value in the cohort; [2] percentages are out of total cases with sufficient data; [3] evaluated by fluorescence in-situ hybridization.

Table S2. Differential gene expression and GSEA comparing low vs. high miR-141/200c-expressing T-PLL.

Table S3. Differential gene expression and GSEA comparing $HuT78^{empty}$ vs. $HuT78^{miR\text{-}141/200c}$ at day 0 or after three days of serum starvation.

Table S4. Differential gene expression and GSEA comparing $HuT78^{empty}$ or $HuT78^{miR\text{-}141/200c}$ at day 0 vs. after three days of serum starvation.

Tables S2–S4 are provided separately, attached as Excel files.

Text S1.

Cell cultures

Suspension cultures of the cell lines HuT78, HH, MOLT-4, SUP-T11, and NK-92 as well as of primary isolates from T-PLL patients and of healthy-donor derived pan-T cells were kept in Gibco™ RPMI-1640 medium supplemented by GlutaMAX™ (ThermoFisher, Waltham, USA) and fetal bovine serum (FBS; 10% for HuT78, MOLT-4, primary T-PLL cells, and healthy-donor pan-T cells; 20% for HH and SUP-T11; 20% + 10 ng/ml Interleukin-2 (ThermoFisher) for NK-92 cells). For serum starvation experiments, the concentration of FBS was reduced to one-tenth. Suspension cells were maintained at a density of $2.0\text{-}8.0x10^5$ cells/ml (cell lines) and of $1.0x10^6$/ml (primary T-cells and T-PLL cells). Cell cultures were kept in an incubator at 37°C and 5% CO2 and 90% humidity. HEK293T cells were cultivated in DMEM medium supplemented with 2mM L-Glutamine and 10% fetal

bovine serum. All cell lines were regularly tested for Mycoplasma infection by standard PCR protocols. The HuT78 cell line was obtained from CLS Cell Lines Service GmbH, Germany, the HH cell line as well as the SUP-T11 cell line from the German Collection of Microorganisms and Cell Cultures GmbH., and the MOLT-4 cell line from the American Type Culture Collection. NK-92 cells were a kind gift from Prof. Satu Mustjoki (Finland, Helsinki). HEK293T cells were a kind gift by Dr. Garry Nolan (Stanford, California, USA)

Plasmid mutagenesis and transfection

To create stable miR-141/200c overexpressing cell lines for *in vitro* experiments on cell proliferation, commercially available lentiviral vectors pLenti 4.1 Ex miR200c-141 and pLenti 4.1 Ex miR200b-200a-429 were purchased[1], both containing a sequence encoding for the green fluorescent protein (GFP). The pLenti Ex miR-200b-200a-429 vector was used as a template for the 'empty' control vector. pLenti Ex miR-200b-200a-429 was digested with restriction enzymes AvrII and EcoRI to remove the genomic sequence for the miR200b/200a/429 cluster. After treatment with AvrII and EcoRI, the remaining plasmid backbone was separated from the miR-200b/200a/429 sequence by agarose gel electrophoresis and extracted using the QIAEX II Gel Extraction kit (Qiaqen, Venlo, Netherlands). Then, an annealed insert of primers containing a four-nucleotide overhang complementary to the restriction sites AvrII and EcoRI, as well as a short, non-coding oligonucleotide sequence containing the recognition site for BamHI was created (forward sequence: CTAGGTAGGGATCCTGAG; reverse sequence: CTCAGGATCCCTACCTAG) to serve as the 'empty' sequence. This 'empty' insert was ligated to the pLenti 4.1 Ex backbone using the LigaFast™ Rapid DNA Ligation System (Promega, Madison, USA) to create pLenti 4.1 Ex empty. Successful ligation of the insert was checked via digestion with the restriction enzyme BamHI. To create competent lentiviral particles containing the pLenti 4.1 Ex miR-200c-141 or empty plasmids, HEK293T were transfected with third-generation lentiviral plasmids pMD2.G, pRSV/Rev and pMDLG/pRRE as well as either pLenti 4.1 Ex miR-200c-141 or empty using TurboFect™ transfection reagent (ThermoFisher, Waltham, USA) according to manufacturer's instructions and cultivated in DMEM medium containing 10% FBS. The supernatant containing competent virus was harvested 48 hours after transfection and subsequently added to the cell lines HuT78, HH, MOLT-4, and SUP-T11 in a ratio of 1:1 virus-containing supernatant to RPMI medium + 30% FBS. Cell cultures were supplemented with Polybrene (8µg/ml). After incubation for 48 hours, growth medium was changed to RPMI + 20% FBS supplemented with 1µg/ml Puromycin. To accelerate the selection process, all cell lines were then FACS-sorted for GFP-positive cells in the core facility of the Max Planck Institute for Biology of Ageing in Cologne using a FACSAria™ Fusion flow cytometer (BD Biosciences, Franklin Lakes, USA; 70 psi pressure, 70 µm nozzle).

Assessment of cell density, viability, proliferation, and mixed culture

For assessment of differences in cell density and viability, cells were seeded at a density of $1x10^5$/ml in a growth medium containing low serum contents (1-2% FBS). Cell density and viability were assessed at the indicated time points via Trypan blue exclusion method using a Countess™ II automated cell counter (ThermoFisher). In order to compare proliferation, cells were stained with eFluor™ 670 Cell Proliferation Dye, a fluorescent dye binding to cellular proteins containing primary amines with subsequent distribution onto daughter cells after cell division, according to the manufacturer's instructions. Median fluorescence intensity (MFI) was measured at the indicated time points via flow cytometry and normalized to values at day 0 to correct for differences in initial staining intensity. The mixed cell culture was set up by mixing unmodified, wild-type (wt) HuT78 cells (GFP-negative) with either $HuT78^{empty}$ or $HuT78^{miR\text{-}141/200c}$ (GFP-positive) in a ratio of 1:1, which was confirmed via flow cytometry. As both $HuT78^{empty}$ and $HuT78^{miR\text{-}141/200c}$ express GFP, the same passage of wt HuT78 cells was used as a surrogate to inquire about competitive growth behavior. Cultures were then seeded at $3x10^5$/ml in RPMI1640 + 1% FBS

and proportions of GFP-positive to GFP-negative cells were determined at the designated time points. The growth medium for mixed cultures was replaced every 3 days.

RNA isolation

Total RNA from T-PLL samples (*n*=55 patients) and CD3+ pan-T cells from six age-matched healthy controls was isolated using the miRVana kit (ThermoFisher, Waltham, USA) according to manufacturer's instructions (Total RNA isolation, no enrichment for small RNAs). DNase treatment using the DNA-free kit (ThermoFisher) was performed to remove remaining DNA residues. Total RNA from cell lines HuT78^{empty} and HuT78$^{miR\text{-}141/200c}$ was isolated using the miRVana kit (ThermoFisher; for qRT-PCR of miR-141/200c) and the RNeasy Plus kit (Qiagen, Venlo, Netherlands) according to manufacturer's instructions. RNA quality and concentration were assessed using the 4150 TapeStation (Agilent, Santa Clara, USA), and samples with an RNA integrity number < 6 were excluded.

RNA sequencing, data processing, and data analysis

We previously subjected RNA from PBMCs of T-PLL patients and CD3^{+} pan-T cells from six age-matched healthy controls to library preparation and polyA-RNA sequencing (*n*=50 T-PLL) as well as small-RNA sequencing (*n*=46 T-PLL). Here, we refer to the previously described procedures regarding RNA isolation, sequencing as well as data processing[2]. For the lentivirally modified cell lines HuT78^{empty} and HuT78$^{miR\text{-}141/200c}$, isolated RNA from *n*=3 basal samples and *n*=3 samples after 3 days of serum starvation was subjected to the library preparation and sequenced on the Illumina TruSeq platform (Illumina, San Diego, USA) according to manufacturer's instructions for polyA-RNA sequencing. Gene set enrichment analysis (GSEA) was performed using the R-package clusterProfiler (v 4.1.4) [3] and MSigDB gene sets (v7.4) [4] utilizing the fgsea algorithm and setting the exponent parameter to 0 for unweighted analyses of log2 fold change sorted gene lists obtained from differential gene expression analyses.

MicroRNA target prediction

The prediction strategy for putative mRNA targets for the miR-141/200c cluster in T-PLL samples was described previously[2]. In short, putative miR targets were mRNAs that were predicted to have miR-141/200c binding sites in at least 2 out of 8 databases (diana_microt, elmmo, microcosm, miranda, mirdb, pictar, pita, targetscan) using the R-package multiMiR (v.1.6.0)[5], as well as a negative Spearman correlation (rho < 0, $p < 0.05$) of their gene expression values to the respective miR expression. For HuT78$^{empty\,/\,miR\text{-}141/200c}$, miR targets were those mRNAs that were predicted as putative targets in at least two databases and were significantly lower expressed in HuT78^{empty} vs. HuT78$^{miR\text{-}141/200c}$ (fold change (fc) < 0.5, FDR < 0.01).

Western blot

Western Blots on whole-cell protein lysates were performed according to standard protocols. The primary antibodies used were STAT4 (cs-2653; Cell Signaling Technology, Danvers, USA) and β-Actin (sc-1616; Santa Cruz Biotechnology, Dallas, Texas) at a 1:1000 dilution. For peroxidase-labeled secondary antibodies we used: anti-mouse (sc-2314), and anti-rabbit (sc-2313; both from Santa Cruz Biotechnology). Chemiluminescence was detected using Autoradiography Film Blue (Santa Cruz Biotechnology) and the developer machine CAWOMAT 2000 IR (CAWO Solutions, Schrobenhausen, Germany).

Quantitative real-time PCR

To confirm the overexpression of miR-141-3p and miR-200c-3p in the lentivirally modified cell lines, isolated RNA was reverse-transcribed into cDNA using the TaqMan™ Advanced miRNA cDNA synthesis kit (Thermofisher). TaqMan™ Advanced miRNA Assays for miR-141-3p, miR-200c-3p, and miR-30c-5p (Thermofisher) were used for quantitative real-time PCRs according to the manufacturer's instructions. Thermal cycling and

detection were carried out using an ABI 7500 Fast System. MiR-30c-5p was used as the endogenous control. Relative quantification was calculated by using the $2^{-\Delta\Delta CT}$ method.

References

1. Gregory, P.A.; Bert, A.G.; Paterson, E.L.; Barry, S.C.; Tsykin, A.; Farshid, G.; Vadas, M.A.; Khew-Goodall, Y.; Goodall, G.J. The MiR-200 Family and MiR-205 Regulate Epithelial to Mesenchymal Transition by Targeting ZEB1 and SIP1. *Nat. Cell Biol.* **2008**, *10*, 593–601. https://doi.org/10.1038/ncb1722.
2. Braun, T.; Glass, M.; Wahnschaffe, L.; Otte, M.; Mayer, P.; Franitza, M.; Altmuller, J.; Hallek, M.; Huttelmaier, S.; Schrader, A.; et al. Micro-RNA Networks in T-Cell Prolymphocytic Leukemia Reflect T-Cell Activation and Shape DNA Damage Response and Survival Pathways. *Haematologica* **2022**, *107*, 187. https://doi.org/10.3324/haematol.2020.267500.
3. Yu, G.; Wang, L.-G.; Han, Y.; He, Q.-Y. ClusterProfiler: An R Package for Comparing Biological Themes among Gene Clusters. *OMICS* **2012**, *16*, 284–287. https://doi.org/10.1089/omi.2011.0118.
4. Liberzon, A.; Birger, C.; Thorvaldsdottir, H.; Ghandi, M.; Mesirov, J.P.; Tamayo, P. The Molecular Signatures Database Hallmark Gene Set Collection. *Cell Syst.* **2015**, *1*, 417–425. https://doi.org/10.1016/j.cels.2015.12.004.
5. Ru, Y.; Kechris, K.J.; Tabakoff, B.; Hoffman, P.; Radcliffe, R.A.; Bowler, R.; Mahaffey, S.; Rossi, S.; Calin, G.A.; Bemis, L.; et al. The MultiMiR R Package and Database: Integration of MicroRNA–Target Interactions along with Their Disease and Drug Associations. *Nucleic Acids Res.* **2014**, *42*, e133. https://doi.org/10.1093/nar/gku631.
